# Serological, Molecular Prevalence and Genotyping of *Coxiella burnetii* in Dairy Cattle Herds in Northeastern Algeria

**DOI:** 10.3390/vetsci9020040

**Published:** 2022-01-22

**Authors:** Salah Eddine Menadi, Valentina Chisu, Cinzia Santucciu, Marco Di Domenico, Valentina Curini, Giovanna Masala

**Affiliations:** 1 Research Laboratory Management of Local Animal Resources, High National Veterinary School “RABIE BOUCHAMA”, Street Issad Abbes, Oued Smar, Algiers 16270, Algeria; s.menadi@etud.ensv.dz; 2 Zoonotic Pathology and OIE Reference Laboratory for Echinococcosis, National Reference Center for Echinococcosis (CeNRE), Istituto Zooprofilattico Sperimentale della Sardegna, 07100 Sassari, Italy; cinzia.santucciu@izs-sardegna.it (C.S.); giovanna.masala@izs-sardegna.it (G.M.); 3 Istituto Zooprofilattico Sperimentale dell’Abruzzo e del Molise G. Caporale, 64100 Teramo, Italy; m.didomenico@izs.it (M.D.D.); v.curini@izs.it (V.C.)

**Keywords:** *Coxiella burnetii*, Q fever, coxiellosis, bulk tank milk (BTM), ELISA, PCR, genotyping, multispacer sequence typing (MST), Algeria

## Abstract

In Algeria, data on the epidemiology of coxiellosis in cattle are still lacking. In this study, bulk tank milk (BTM) samples from 200 randomly selected dairy cattle herds from Setif province of Algeria were analyzed by an indirect enzyme-linked immunosorbent assay (ELISA) and polymerase chain reaction (PCR). Results highlighted that 37% (95% CI: 30.31–43.69%) and 9% (95% CI: 5.03–12.96%) of BTM samples contained *Coxiella burnetii* antibodies and DNA, respectively. Based on Cohen’s kappa coefficient, a very low agreement between the ELISA and PCR results was found (k = 0.0849) (95% CI: 0.00–0.189). For a second experiment, 186 whole blood samples of cows from farms with reproduction disorders were analyzed by molecular tools to detect C. burnetii. This study revealed an overall prevalence of 6.98% (95% CI: 3.32–10.65%). All positive samples determined by conventional PCR were analyzed by real-time quantitative PCR (qPCR). Eleven samples with cycle threshold (Ct) values lower than 35 were selected for genotyping by the multispacer sequence typing (MST) method. The MST12 genotype in BTM samples, the MST32 genotype and a new MST genotype (partial profile) in whole blood samples were identified. Obtained results have allowed us to better understand the epidemiology of bovine coxiellosis in the region of Setif.

## 1. Introduction

*Coxiella burnetii* is a zoonotic intracellular bacterium that has a wall similar to that of gram-negative bacterium, but it is not stainable by the Gram stain [1]. It causes Q fever in humans and coxiellosis in animals [2]. This disease has economic importance and an impact on global public health since it is a ubiquitous zoonosis with the exception of New Zealand. *C. burnetii* belongs to the Coxiellaceae family, Legionellales order of the gamma subdivision of Proteobacteria [3]. It can infect various mammals, non-mammals, vertebrates and ticks [4]. However, ruminants are the main reservoir of human infections [5]. Coxiellosis is often asymptomatic in domestic ruminants; however, in goats and ewes, this infection could manifest as late-term abortion, stillbirth and the delivery of weak offspring [6]. In cattle, it may be associated with endometritis, infertility and mastitis [2]. Q fever in humans may be asymptomatic, acute or chronic. The acute form manifests with fever, atypical pneumonia and hepatitis; the chronic type presents long-term sequelae, including fatigue, abortions and heart disease [7]. Both animals and humans could be infected through the inhalation of contaminated aerosols generated from the excreta of infected animals [8]. *C. burnetii* presence was reported in birth products during and after parturition or abortion, urine, faeces, vaginal discharges and milk related to both symptomatic and asymptomatic infected ruminants [9]. This agent can be excreted into the milk for 8 days in ewes and up to 13 months in cows [10]. However, according to several authors, the contamination by oral route remains controversial [11,12]. Other transmission routes may be the ingestion of contaminated animal products or even tick bites [13,14]. Finally, *C. burnetii* is extremely resistant, remaining viable in the environment over long periods [15] in hot and dry weather conditions [10,16].

The analysis of bulk tank milk (BTM) samples was previously used for monitoring coxiellosis in dairy cattle herds [17,18]. BTM sampling has several advantages. Since it provides a representative sample for all lactating cows, it is easy to obtain, non-invasive, convenient and economic [18,19]. The polymerase chain reaction (PCR) and the enzyme-linked immunosorbent assay (ELISA) methods are the most used assays to perform diagnosis on *C. burnetii* at herd level in most of the epidemiological investigations conducted around the world [20]. Indeed, the serological analysis of BTM samples by ELISA test for antibodies against *C. burnetii* detection, reveals the possible previous exposure of herds. However, PCR detects *C. burnetii* shed in milk and thus reveals the current infection [21,22]. Genotypic characterization of *C. burnetii* isolates can improve the ability to identify a source of infection, helps to establish prevention and control measures, reduces the number of cases during an outbreak [23], decreases malicious use of this bacteria and helps to identify and track the virulent lineage [24]. Whole-genome sequences have enabled the application of a range of highly discriminatory typing approaches. Multispacer sequence typing (MST) genotyping is based on the study of the variation of sequences of 10 intergenic regions (spacers) located between two open reading frames (ORFs) [23,25]. Similar to the multiple-locus variable analysis (MLVA) genotyping method, MST is known to be reliable, reproducible and very discriminating, not requiring prior culture of *C. burnetii* under biosafety level 3 conditions and can be implemented directly on the extracted DNA from clinical and environmental samples [26]. According to recent reviews [20,27], there are no studies concerning the presence of *C. burnetii* in BTM samples of cows in all Africa, and only one study described *C. burnetii* in individual bovine milk samples in Egypt [28]. However, some epidemiological studies on the seroprevalence of coxiellosis in cattle [29,30,31,32,33] and Q fever in humans have been reported in Algeria [34]. Furthermore, the genotypic characterization of circulating *C. burnetii* strains in Algeria is pivotal to increasing the richness of molecular epidemiology data for Q fever. The goal of this study was to estimate the prevalence, the infection rate and the genotype of *C. burnetii* from cattle in farms with reproduction disorders in the Setif region of Algeria, Africa.

## 2. Materials and Methods

### 2.1. Study Area

**Figure 1:**
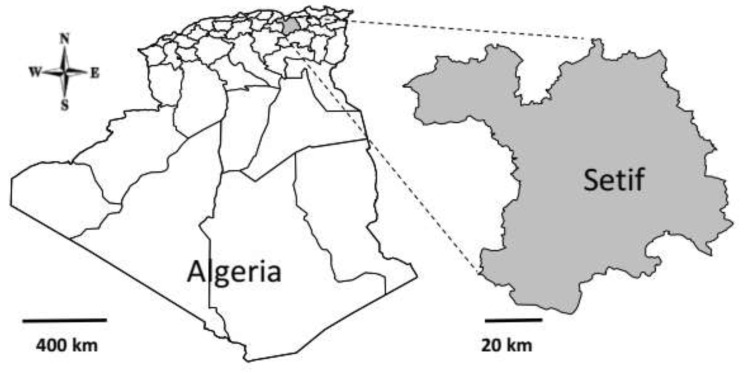
Map of the state of Setif in northeastern Algeria where samples were collected during the period from September 2017 to April 2018 for the detection of *C. burnetii* infection in cattle.

This study was conducted between September 2017 and April 2018 in Setif province. It is one of the 48 provinces of Algeria with a surface area of 6550 km^2^, covering 0.27% of the total area of Algeria. It is located in the northeast of the country (latitude, 35°61′–36°59′ N; longitude, 4°73′–6°02′ E) (Figure 1). The climate is semi-arid Mediterranean, characterized by cold rainy winters and hot, dry summers. January is the coldest month with a mean temperature of 5.03 °C, while July is the hottest month with a mean temperature of 26.07 °C.

### 2.2. Sampling

All specimens of milk and blood investigated in this study were taken in the Setif area, Algeria, Africa. Firstly, in this cross-sectional study at the herd level, the sample size was determined by the formula for simple random sampling proposed by Thrusfield [35]:

N = (1.96)^2^ P (1 − P)/L2

where N is the sample size, 1.96 is the Z value for the selected confidence level (95%), P is the expected disease prevalence and L is the desired absolute precision. A minimum sample size of 96 dairy herds was obtained using an expected herd prevalence of 50%; since there were no previous studies in the Setif area, a desired absolute precision of 10% and a confidence interval of 95% were required.

We decided to collect one bovine BTM sample from each commercial dairy herd that contained milk from at least 10 lactating cows after obtaining verbal consent from the farmers. In the cases in which the farmer refused, we were involved in sampling the closest neighbouring herd. To increase the precision, a total of 200 dairy herds were randomly selected among the 3900 existing in the Setif area. The resulting 200 BTM samples collected were mixed into each tank, then aliquoted into sterile 50 ml plastic tubes, transported on ice to the testing laboratory and promptly stored at −20 °C until use.

Moreover, an additional 186 whole blood samples were collected from 26 cattle herds with reproductive disorders (history of abortion, neonatal mortality and infertility) and whose milk was not sampled to detect the presence of *C. burnetii* in dairy cows in this region. Blood samples were collected from the coccygeal vein of each cow into EDTA tubes using disposable needles and plain vacutainer tubes and transported on ice to the laboratory. EDTA tubes were directly frozen at −20 °C until use.

### 2.3. Laboratory Analysis

The laboratory analyses of the samples were performed at Istituto Zooprofilattico Sperimentale della Sardegna, Sassari, Italy.

#### 2.3.1. Serological Analysis

Whole BTM samples from each herd, previously aliquoted in 50 mL tubes, were tested for *C. burnetii* antibodies using the indirect commercial ELISA kit PrioCHECK Ruminant Q Fever Ab Plate Kit (LSI, Lissieu, Lione, France), formerly LSIVet Ruminant Q Fever Serum/Milk. The principle of the test is based on the use of inactivated *C. burnetii* phase 1 and phase 2 antigens obtained from the ovine strain of *C. burnetii* (CbO1) that is responsible for ovine abortion. Samples were considered positive when more than 10% of lactating cows in the herd had specific antibodies against *C. burnetii* [18,36,37,38]. In addition, the assay was validated to be used as a bulk milk test by Muskens et al., 2011 [18]. Samples analysis and results interpretation were performed according to the manufacturer’s instructions. Optical density (OD) values were measured at 450 nm, and sample/positive percentages (S/P%) of each sample were calculated according to the formula:

S/P% = 100*(OD sample − OD negative control)/(OD positive control − OD negative control). Samples were considered negative for a S/P% ≤ 30%, low positive for a 30 < S/P% ≤100% (+), positive 100< S/P% ≤200% (++) and high positive for S/P% > 200% (+++). 

#### 2.3.2. Molecular Analysis

*DNA extraction.* All 200 BTM samples were pre-treated before DNA extraction as follows: after thawing and mixing, 1.5 mL BTM were distributed into a 2 mL Eppendorf tube, then centrifuged at 10,000 rpm for 10 min at 4 °C. The top layers of the cream and the sera were removed. Instead, the cell pellet was washed twice with phosphate-buffered saline (PBS) 1× and once with Sodium Dodecyl Sulfate (SDS) 1%, then diluted with PBS 1×. Each washing step was followed by centrifugation at 10,000 rpm for 10 min at 4 °C and removal of the supernatant. The 186 blood samples were undergone to total genomic DNA extraction, along with those of BTM, using the commercial DNeasy Blood & Tissue kit (Qiagen, Hilden, Germany). 

*Amplification:* The genomic DNAs were stored at −20 °C until use. The IS1111a partial sequence of 154 base pairs (bp) was amplified using the primers named IS1111F 5′-CAAGAAACGTATCGCTGTGGC-3′ and IS1111R 5′ CACAGAGCCACCGTATGAATC-3′ [39]. The PCR was performed on 25 μL of total reaction volume including 9.5 μL of H_2_O milliQ RNasy-free, 12.5 μL of Mix Quantitech (Qiagen, Hilden, Germany), 1 μL of each primer (1 pM) and 1 μL of the DNA extracted from each sample. Positive controls (prepared from the placenta of an aborted sheep infected with *C. burnetii*) and negative controls (H_2_O MilliQ water) were added at each run. DNA amplification was performed in an automatic SimpliAmp thermocycler (Thermofisher Scientific, Waltham, MA, USA) with an activation step at 95 °C for 15 min, followed by 40 cycles of denaturation at 94 °C for 1 min, annealing at 62 °C for 30 s, extension at 72 °C for 1 min and a final extension at 72 °C for 5 min. PCR products were visualized by electrophoresis on a 1.5% agarose gel, stained with SYBR Safe DNA Gel Stain (Invitrogen, Carlsbad, CA, USA) and examined using an ultraviolet transilluminator.

*Sequencing*: In order to confirm the PCR results, all amplicons were purified using the QIAquick Spin PCR Purification Kit (Qiagen, Hilden, Germany) and sequenced using the Big Dye Terminator cycle sequencing ready reaction kit (Thermofisher Scientific, Waltham, MA, USA) using a 4 capillary electrophoresis Genetic Analyzer ABI 3130 (Thermofisher Scientific). The obtained sequences were analyzed using the ABI PRISM DNA Sequencing Analysis software version 3.0 (Thermofisher Scientific, Waltham, MA, USA), assembled and edited using ChromasPro software version 2.2 (Technelysium Pty Ltd., Tewantin, QLD, Australia) and compared to the sequences available in the GenBank database using the BLAST algorithm (http://blast.ncbi.nlm.nih.gov/Blast.cgi, accessed on 18 May 2018). 

*Real-time PCR:* All positive samples were also analyzed by quantitative real-time PCR (qPCR), a method recently set up in our laboratory by means of a commercial kit, the VetMAX™ *C. burnetii* Absolute Quant Kit (LSI, Lissieu, France) employed on a 7500 Fast Real-Time PCR Instrument (Thermofisher Scientific, Waltham, MA, USA).

*Genotyping*: Positive samples showing cut-off cycle threshold values below 35 (Ct < 35) were sent to the laboratory of the Istituto Zooprofilattico Sperimentale dell’Abruzzo e del Molise, Teramo, Italy, for genotyping by MST. Ten selected intergenic spacers from the *C. burnetii* genome (Cox2, 5, 18, 20, 22, 37, 51, 56, 57 and 61) were amplified and sequenced according to the protocol of Glazunova et al., 2005 [25], with some modifications [40].

### 2.4. Statistical Analysis

Seroprevalence of the herds, determined by the presence of antibodies against *C. burnetii* in milk, was calculated from the ratio of positive herds to the total number of herds investigated with the exact binomial confidence interval of 95% [35]. Then, to evaluate the agreement between the results obtained by serological and molecular results performed on the 200 BTM samples, Cohen’s Kappa test was employed [41]. The K value was classified into classes [42]. Agreement is considered as: almost perfect (0.81 ≤ K ≤ 1), substantial (0.61 ≤ K≤ 0.80), moderate (0.41≤K≤0.60), fair (0.21 ≤ K ≤ 0.40), slight (0.00 ≤ K ≤ 0.20), poor (K < 0.00). Finally, the McNemar test was also used to compare the proportion of positive results between these two diagnostic methods [43] performed on the 200 BTM samples. The statistical analysis was performed using SPSS v25.0 software (SPSS Inc., Chicago, IL, USA).

## 3. Results

### 3.1. Serological Analysis

Serological analysis of the 200 BTM tested by ELISA evidenced 74 herds positive to the presence of anti-*C. burnetii* antibodies (37%; 95% CI: 30.31%–43.69%). Three levels of OD intensity were determined for all herds involved in the study, according to the manufacturer’s instructions. In detail, a total of 54 BTM samples were found lowly positive (+) (27%; 95% CI: 20.84–33.15%) and 20 presented a positive result on average (++) (10%; 95% CI: 5.84–14.15%), while none of the herds presented highly positive results (+++).

### 3.2. Molecular Analysis

The PCR molecular analysis of the 200 BTM samples was able to evidence 18 samples (9%; 95% CI: 5.03–12.96%) positive for *C. burnetii* DNA. Secondly, the PCR of the 186 blood samples from 26 herds with reproductive disorders evidenced 13 cows positive for IS1111 from 6 different farms, which corresponds to an individual infection rate of 6.98% (95% CI: 3.32–10.65%) and a herd infection rate of 23.07% (95% CI: 6.88–39.27%).

### 3.3. Sequencing

BLASTn (https://blast.ncbi.nlm.nih.gov/Blast.cgi?PAGE_TYPE=BlastSearch, accessed on 18 May 2018) analysis confirmed the *C. burnetii* DNA detection by PCR. Moreover, the IS1111 sequences obtained from bovine milk showed 100% identity with those isolated from blood samples and those of *C. burnetii* retrieved from the Genbank database (accession number CP035112.1, CP040059.1, CP014354.1, CP013667.1). 

### 3.4. Statistical Analysis

The global prevalence of the 200 BTM obtained by both ELISA and PCR assays corresponded to 41% 95% CI: 34.18%–47.82%. Coupling the results obtained by the *C. burnetii* DNA detection and its antibodies, performed respectively by PCR and ELISA, a total of 10 samples turned positive by both assays (5%; 95% CI: 1.98–8.02%). On the other hand, 8 samples tested positive by only PCR (4%; 95% CI: 1.28–6.71%), 64 samples were positive by only ELISA (32%; 95% CI: 25.53–38.46%), while 118 samples were negative by both assays (59%; 95% CI: 52.18–65.81%) (Table 1). Cohen’s kappa test showed a coefficient k corresponding to 0,0849 (95% IC: 0–0.189), which corresponds to a slight agreement between the ELISA test and PCR results. Furthermore, the McNemar test showed that both assays gave significantly different results (*p* < 0.01) (Table 1).

### 3.5. Genotyping

Four BTM samples and seven whole blood samples, with Ct values lower than 35, were genotyped by MST method. Only one whole blood sample and two BTM samples with Ct values less than 29 gave a complete MST genotyping profile, where all intergenic spacers were successfully amplified. Regarding the remaining eight samples, we observed a positive result by PCR of at least three intergenic spacers (Table 2).

The combination of the alleles of the intergenic spacers obtained revealed the presence of two genotypes, the MST12 in the BTM samples and the MST32 in the blood samples (Table 2). A new MST genotype (partial profile) was also identified in the blood samples of two cows belonging to the same farm (farm 2) with allele codes of 3-5-5-5-1-6-5 for the Cox2-Cox5-Cox18-Cox22-Cox37-Cox57-Cox61 spacers, respectively (Table 2).

## 4. Discussion

This work is the first cross-sectional study in which BTM samples from the Setif region, Algeria, were screened to search for antibodies against *C. burnetii;* moreover, molecular typing was employed to identify the circulating strains of *C. burnetii*. The ELISA kit used in this study was based on *C. burnetii* antigens isolated from ruminants, and it was found to likely be more sensitive than the ELISA kit based on *C. burnetii* Nine Mile strain, isolated from ticks [44]. In total, 37% of BTM samples tested positive for *C. burnetii* antibodies after ELISA analyses. Before that, a seroprevalence of 45% was reported from sera samples in the same region [33]. The decrease in seroprevalence values observed (that resulted lower than that previously obtained) could be related to the improvement of standard hygienic measures in industrial livestock taken to prevent *C. burnetii* environment dissemination. In addition, the serological criteria used to evaluate *C. burnetii* seroprevalence in cattle herds varied according to the diagnostic matrix used (a single cow or BTM). Indeed, in the previous study, a bovine herd was considered seropositive when it contained at least one seropositive cow [33], while in the present study, the herd was considered seropositive when it contained at least 10% of seropositive lactating cows. The sensitivity of this ELISA kit decreases when the intra-herd seroprevalence is less than 10% [18,36,37,38]. In addition, tank milk samples do not include dry or sick cows (e.g., mastitis), which may possibly be seropositive. The exhaustive literature, which highlights that the seroprevalence of *C. burnetii* in herd cattle is highly variable worldwide. The prevalence rate of *C. burnetii* from BTM samples here obtained was similar to those reported in Portugal (37.8%) [38], but lower than that observed in Poland (45.5%) [45], Belgium (57.8%) [37], Denmark (59%) [46], Spain (66.9%) [47], Netherlands (78.6%) (81.6%) [18,48] and Jordan (70.9%) [49]. 

The prevalence of *C. burnetii* DNA is also lower than those found in The Netherlands (56.6% and 18.8%) [18,48], Portugal (20%) [38], Belgium (30%) [37], Poland (36.9%) [45], Spain (51.7%) [47] and the United States (94.3%) [17]. This could be explained by the high number of bovine herds that could favour the spread of *C. burnetii* infection between animals in these countries [38,48,50,51]. Moreover, the choice of sampling strategy and the tools used for the diagnosis of coxiellosis [52] could determine differences in prevalence rates from one country to another. 

However, to improve serological diagnosis, the real rate of coxiellosis in herds was achieved by adding PCR analyses on BTM. In our study, 9% of the pooled samples analyzed resulted positive after PCR analyses targeting the transposase gene of insertion element IS1111. Among the PCR-based assays developed for *C. burnetii* detection in clinical samples, the IS1111 target allows highly sensitive detection of *C. burnetii* DNA since several copies of this gene are present in the genome [53,54]. Importantly, vaccination against *C. burnetii* is not carried out in Algeria, so our results reflect the natural infection. Cohen’s kappa-based test showed a slight agreement between ELISA and PCR results from testing bovine BTM with a K value of 0.0849 (95% CI: 0–0.189). Furthermore, the McNemar test revealed that both assays gave significantly different results (*p* < 0.001) in agreement with other studies [37,38]. This discrepancy could be related to several aspects. ELISA-positive and PCR-negative (32%) indicated a past *C. burnetii* infection with the absence of bacterial shedding in cows. Conversely, the presence of PCR positive samples and ELISA negative (4%) could be explained by a recent infection of *C. burnetii* in cows in which antibodies are not yet produced against the bacteria, or the level of these is below the threshold of detectability [18,38]. In fact, a comparison between molecular and serological methods to diagnose *C. burnetii* in milk is not appropriate because the immunological response is longer than the shedding of this bacteria into milk [55]. Moreover, *C. burnetii* shedding was sometimes intermittent when occurring in seronegative animals [56], and can also occur via other routes such as vaginal mucus and feces and not in the milk [21]. In this study, we found an infection rate of 6.98% in cows from farms with a history of reproductive disorders. Several studies have speculated that *C. burnetii* could determine reproductive disorders in ruminants such as infertility [57], abortion [58,59] and neonatal mortality [2,60]. For this reason, whole blood of cows from farms presenting reproductive problems were analyzed in order to increase the detection of *C. burnetii* DNA and perform genotyping. Molecular tools for *C. burnetii* detection are essential for epidemiological investigations carried out during the appearance of *C. burnetii* infection foci or during surveillance. MST and MLVA represent two discriminating methods commonly used for *C. burnetii* genotyping. In this study, MST was used to determine the *C. burnetii* genotypes present in blood samples and in the BTM. This method has been found to be more laborious and less discriminating than the MLVA [23,25]. However, it has the advantage of using a standardized nomenclature and having a database that allows easy comparison of results between laboratories [61]. The MST method identified 30 different genotypes and three monophyletic groups among 173 isolates of *C. burnetii*, based on the combination of the different sequences of 10 intergenic spacers [1,25]. Since then, more than 50 MST groups have been described (https://ifr48.timone.univ-mrs.fr/mst/coxiella_burnetii/strains.html, 03 March 2021). In our study MST genotyping showed the presence of the MST12 in BTM samples and MST32 and a novel MST genotype from cattle blood samples. This correlation between the nature of the sample and the MST genotype could be related to the low number of highly concentrated samples of *C. burnetii* DNA that have succeeded in genotype determination by MST. The MST12 and MST32 genotypes belong to the monophyletic group II and are closely related on the basis of phylogenetic analysis [62]. The MST12 genotype has been detected in human clinical samples (heart valve, human blood, retrosternal abscess, aneurysm, valve prosthesis and spleen abscess) in France, Switzerland and Senegal (https://ifr48.timone.univ-mrs.fr/mst/coxiella_burnetii/strains.html, 03 March 2021), while in animals, it has been found in sheep cheese in Italy [63], and in samples of lung, spleen, vaginal swabs and placentas collected from small ruminants in Italy [64]. However, to the best of our knowledge, this is the first time that the MST12 genotype has been identified in bovine samples. The MST32 genotype was previously detected in human samples (a heart valve in Germany and an aortic biopsy in France), goat placenta in Austria (https://ifr48.timone.univ-mrs.fr/mst/coxiella_burnetii/strains.html, 03 March 2021), sheep cheese in Italy [63], in liver samples, gastric contents, cotyledons sampled from sheep in Greece [64], in spleen, brain, vaginal swabs, lung, liver samples from small ruminants and individual milk from dairy cows in Italy [65]. The MST12 and MST32 genotypes were first described in Algeria. A recent Algerian study highlights the presence of MST20 genotype in placentas of dairy cows having aborted [66]. The presence of these different genotypes can be explained by the importation of animals, in particular cattle and goats from several countries (Netherlands, Germany, France, Spain, Austria, etc.) as well as their movements in neighbouring countries (Tunisia, Niger, Mali, etc.). Finally, the allele codes identified in the new MST genotype are 3-5-5-5-1-6-5 for the Cox2-Cox5-Cox18-Cox22-Cox37-Cox57-Cox61 spacers, respectively. This profile differs considerably from all those of the genotypes described above. Unfortunately, PCR amplification was not successful for all spacers (partial profile) due to the low amount of *C. burnetii* DNA in our blood samples, which made it impossible to describe the full profile of this new MST genotype and upload it to the MST database (https://ifr48.timone.univ-mrs.fr/mst/coxiella_burnetii/strains.html, 03 March 2021). To this end, further studies should be carried out to fully identify this genotype and understand its pathogenic, infectious and zoonotic potential.

## 5. Conclusions

The results of this study showed that 37% and 9% of dairy herds contained *C. burnetii* antibodies and *C. burnetii* DNA, respectively, in the Setif region of Algeria. These rates are relatively low when compared with those published in other countries worldwide. However, the implementation of prophylaxis and control programmes are necessary to avoid the spread of *C. burnetii* infection among cattle and the potential risk for public health. BTM testing is a good tool for coxiellosis monitoring purposes in dairy cattle herds providing diagnosis through the combination of molecular and serological tests and the repetition of the sampling. This study provides information on the genotypic diversity of *C. burnetii* infecting cattle in Algeria. MST genotyping analysis focused on the region of Setif showed for the first time the presence of the MST12, MST32 genotypes and a new MST genotype (partial profile). The MST12 and MST32 genotypes have previously been described in human clinical samples, suggesting that cattle may play an important role as reservoirs for human Q fever infection in Algeria.

## Figures and Tables

**Table 1:** Agreement between the results of the analysis of bovine bulk milk tank (BMT) positive samples by means of two diagnostic methods (ELISA and PCR) for *C. burnetii*.

Test		ELISA		Total
		Positive	negative	18
**PCR**	positive	10	8	
	negative total	64 74	118 126	182 200
Cohen’s kappa: 0.0849 (95% CI: 0.0–0.189)				
Mc Nemar test: 9.06^−11^				

**Table 2:** Results of qPCR quantification and MST genotyping of Coxiella burnetii DNA in cows in Setif region, Algeria.

Identification Code	Farm Code	Type of Sample	Cycle Threshold Value	Intergenic Spacer										MST Genotype
				Cox 2	Cox 5	Cox 18	Cox 20	Cox 22	Cox 37	Cox 51	Cox 56	Cox 57	Cox 61	
1	Farm 1	Whole blood	31.6	NA	NA	NA	NA	5	NA	NA	12	3	NA	NI
2	Farm 1	Whole blood	31.5	3	5	1	NA	5	NA	NA	12	3	NA	MST32
3	Farm 2	Whole blood	30.8	3	5	5	NA	5	NA	NA	NA	NA	5	New
4	Farm 2	Whole blood	30.2	3	5	5	NA	5	1	NA	NA	6	5	New
5	Farm 3	Whole blood	32.3	NA	5	1	NA	5	4	NA	NA	3	NA	MST32
6	Farm 3	Whole blood	32.2	NA	5	1	NA	5	4	NA	12	3	NA	MST32
7	Farm 4	Whole blood	26	3	5	1	6	5	4	5	12	3	2	MST32
8	Farm 5	BMT	32.7	3	NA	NA	NA	5	4	NA	NA	NA	NA	NI
9	Farm 6	BMT	34.9	3	NA	NA	NA	5	4	NA	NA	NA	NA	NI
10	Farm 7	BMT	28.2	3	5	1	6	5	4	5	4	3	2	MST12
11	Farm 8	BMT	28.3	3	5	1	6	5	4	5	4	3	2	MST12

NA: not amplified; 1, 2, 3, 4, 5, 6, 12: The allele codes assigned to each amplified spacer; NI: Not identified.

## Data Availability

The data presented in this study are available on request from the corresponding author.
